# DNA and scale reading to identify repeat spawning in Atlantic salmon: Unique insights into patterns of iteroparity

**DOI:** 10.1111/eva.13612

**Published:** 2023-11-23

**Authors:** Håvard Kaland, Alison Catherine Harvey, Øystein Skaala, Vidar Wennevik, Francois Besnier, Per Tommy Fjeldheim, Sofie Knutar, Kaja Christine Andersen, Kevin Alan Glover

**Affiliations:** ^1^ Institute of Marine Research Bergen Norway; ^2^ Department of Biological Sciences Ålesund Norwegian University of Science and Technology Trondheim Norway; ^3^ Department of Biology University of Bergen Bergen Norway

**Keywords:** alternate repeat spawner, Atlantic salmon, consecutive repeat spawner, DNA profiling, Etne, repeat spawners

## Abstract

Iteroparity represents an important but often overlooked component of life history in anadromous Atlantic salmon. Here, we combined individual DNA profiling and scale reading to identify repeat spawners among ~8000 adult salmon captured in a fish trap in the river Etne, Norway, in the period 2015–2019. Additionally, 171 outward migrating kelts were captured in the spring of 2018–2020 and identified using molecular methods to estimate weight loss since ascending the river to spawn. The overall frequency of repeat spawners identified using molecular methods and scale reading combined was 7% in females and 3% in males (5% in total). Most of these (83%) spent one full year reconditioning at sea before returning for their second spawning, with a larger body size compared with their size at first spawning, gaining on average 15.9 cm. A single female migrating back into the river for a fifth breeding season was also identified. On average, kelts lost 40% bodyweight in the river, and more female than male kelts were captured during outward migration. The date of arrival in the upstream fish trap was significantly but moderately correlated between maiden and second entry to the river for alternate and consecutive spawners. The estimated contribution from repeat spawners to the total number of eggs deposited in the river each year varied between 2% and 17% (average 12%). Molecular‐based methods marginally underestimated the number of repeat spawners compared with scale reading (5% vs 7%) likely due to a small number of returning spawners not being trapped and sampled. Differences between the methods were most evident when classifying the spawning strategy (alternate or consecutive‐year repeat spawners), where the scale method identified proportionally more consecutive‐year repeat spawners than the molecular method. This unique data set reveals previously unstudied components of this life history strategy and demonstrates the importance of repeat spawners in population recruitment.

## 1 INTRODUCTION

The trade‐off between survival and reproductive success resulting in a species being either semelparous or iteroparous has long been expounded (Bordeleau et al., 2020, Seamons & Quinn, 2010, Stearns, 1976). If adult survival is low relative to offspring survival, then withholding additional energic resources for future reproduction, that is, iteroparity, is not the favoured reproductive strategy, resulting in semelparity, where an individual reproduces in one massive fatal event. However, if reproductive success or offspring survival are too variable to risk in one event, iteroparity will be favoured, where the reproductive contribution of an individual is spread across multiple events over time. While most animals display iteroparous mating systems, semelparity is observed in a handful of mammals, many invertebrates and plants and some fish species (Fisher et al., 2013). Fish in the Salmonidae family fall into both categories, with most in the genus *Oncorhynchus* known for semelparous reproduction and those in the *Salmo* and *Salvelinus* genera being known for iteroparity (Fleming, 1998).

Atlantic salmon (*Salmo salar L*.) display some of the most diverse life‐history strategies in the world (Erkinaro et al., 2019), many of which are locally adaptive and may involve evolutionary mechanisms such as natural selection (Garcia de Leaniz et al., 2007). It is one of the most studied salmonids, with most populations displaying an anadromous life history. Juveniles typically remain in the river for 2–4 years before migrating to the ocean to feed for a further 1–3 years before returning to their native rivers to reproduce. Fish that survive spawning, commonly called kelts, will migrate back to the sea either immediately after spawning or overwinter in the river and migrate back to sea the following spring. Thereafter, survivors may return to the river to spawn again, having spent either less than 1 year reconditioning at sea (consecutive repeat spawners) or after one or more years at sea (alternate repeat spawners) (Birnie‐Gauvin et al., 2019; Chaput & Benoit, 2012; Harvey et al., 2022). However, kelt survival at sea is expected to be low (Jonsson, Hansen, & Jonsson, 1991; Klemetsen et al., 2003), with the energetic costs of spawning and the variable environmental conditions experienced by spent spawners affecting their ability to recondition and return as repeat spawners (Bordeleau et al., 2020; Chaput & Benoit, 2012). Studies report a variability of between 2% and 25% in the proportion of kelts returning to spawn (Bøe et al., 2022; Halttunen, 2011; Jonsson, Hansen, & Jonsson, 1991; Reddin et al., 2011). The survival of both in‐ and out‐migrating salmon is also negatively affected by anthropogenic effects, such as river barriers or exploitation (Erkinaro et al., 2019).

The frequency of repeat spawners observed in salmonid populations is highly variable between rivers and regions. Fleming (1998) found that Atlantic salmon repeat spawners constitute on average 11% of a given population (range 1%–43%), which is within the lower range of observed levels of repeat spawners in other iteroparous salmonid species. Persson et al. (2022) looked at incidence of iteroparity in 179 salmon populations in rivers in Norway and found that rivers in the south were more likely to contain fish that spawned more than once than rivers in the north of Norway. Bordeleau et al. (2020) examined the incidence of iteroparity in 10 populations of Atlantic salmon in the northwest Atlantic and found that the average incidence of repeat spawners was 5% (range 0–24.7%). However, they reported that trends diverged over time, with increases in iteroparity in the mid and northern populations and decreases in the south. Both increases and decreases in iteroparity over time have been documented in other studies in Atlantic salmon (Erkinaro et al., 2019; Maynard et al., 2018).

Females are often more abundant as repeat spawners than males and may contribute significantly to recruitment through their high fecundity which is linked to body size (Halttunen, 2011; Harvey et al., 2022; Niemelä, Erkinaro, et al., 2006; Reid & Chaput, 2012). Skewed sex ratios among repeat spawners are often observed in salmonids, including Atlantic salmon and steelhead trout (*Oncorhynchus mykiss W*.), and it is hypothesized that this is due to competition and behaviour differences between the sexes on the spawning grounds (Fleming, 1998; Seamons & Quinn, 2010) or different energy expenditures related to reproduction and influencing reconditioning between the sexes (Persson et al., 2022). Christie et al. (2018) found that negative frequency‐dependent selection was responsible for older, larger female repeat spawners being more productive when they were less abundant than younger, smaller females and males. Repeat spawners follow an alternate or consecutive repeat spawning strategy (Halttunen, 2011; Persson et al., 2022). Spending one more year in the ocean before returning to the river to spawn allows for more time to recondition and regain weight, potentially a strategy for maximizing fecundity and survival in the next spawning event. Repeat spawning strategies may also be size dependent, with smaller fish able to recover enough to return to spawn annually, while larger fish need more time to restore depleted energy reserves (Bøe et al., 2022).

Iteroparity can provide a buffer against unpredictable events that jeopardize population recruitment and may be a valuable source of genetic variability (Harvey et al., 2022; Narum et al., 2008; Reid & Chaput, 2012). Understanding the extent to which repeat spawners occur in a population is therefore vital for population management and estimation of sustainable exploitation rates. Despite this, iteroparity remains an understudied aspect of salmonid life history (Bordeleau et al., 2020). Those studies that have examined iteroparity in Atlantic salmon have utilized various methods to quantify and describe the frequency and demography of the repeat spawners in a population. The most popular methods are tagging‐based studies and/or classification based on scale reading (Bøe et al., 2022; Chaput & Benoit, 2012; Erkinaro et al., 2019; Halttunen, 2011). Tagging allows for individual data usually based on a subsample of a population but may cause handling stress and increased mortality of tagged individuals and possible underreporting of tagged fish that are recaptured (Thorstad et al., 2013). Scale analysis, where a scale from a fish is examined under a microscope for tell‐tale spawning marks (Seamons et al., 2009), can yield data on potentially more fish, although this method depends on good quality scales and cannot yield individual data on size or timing of river entry at previous spawning time points. More recently, molecular tools have been used to identify repeat spawners in populations based on tissue samples taken at different time points or through parentage and mixed‐stock fishery assignment (Christie et al., 2018; Seamons & Quinn, 2010). The potential advantages of combining the two approaches are the ability to include information on the previous spawning events of individuals, for example, on change in size and on the timing of subsequent migrations coupled with demographic details such as age at first maturity, and potentially including more individuals than in tagging studies. To our knowledge, comparisons between genetic methods and scale analysis are rare (but see Seamons et al., 2009), and the complimentary use of molecular methods and scale reading has not been used in Atlantic salmon to date.

**FIGURE 1:**
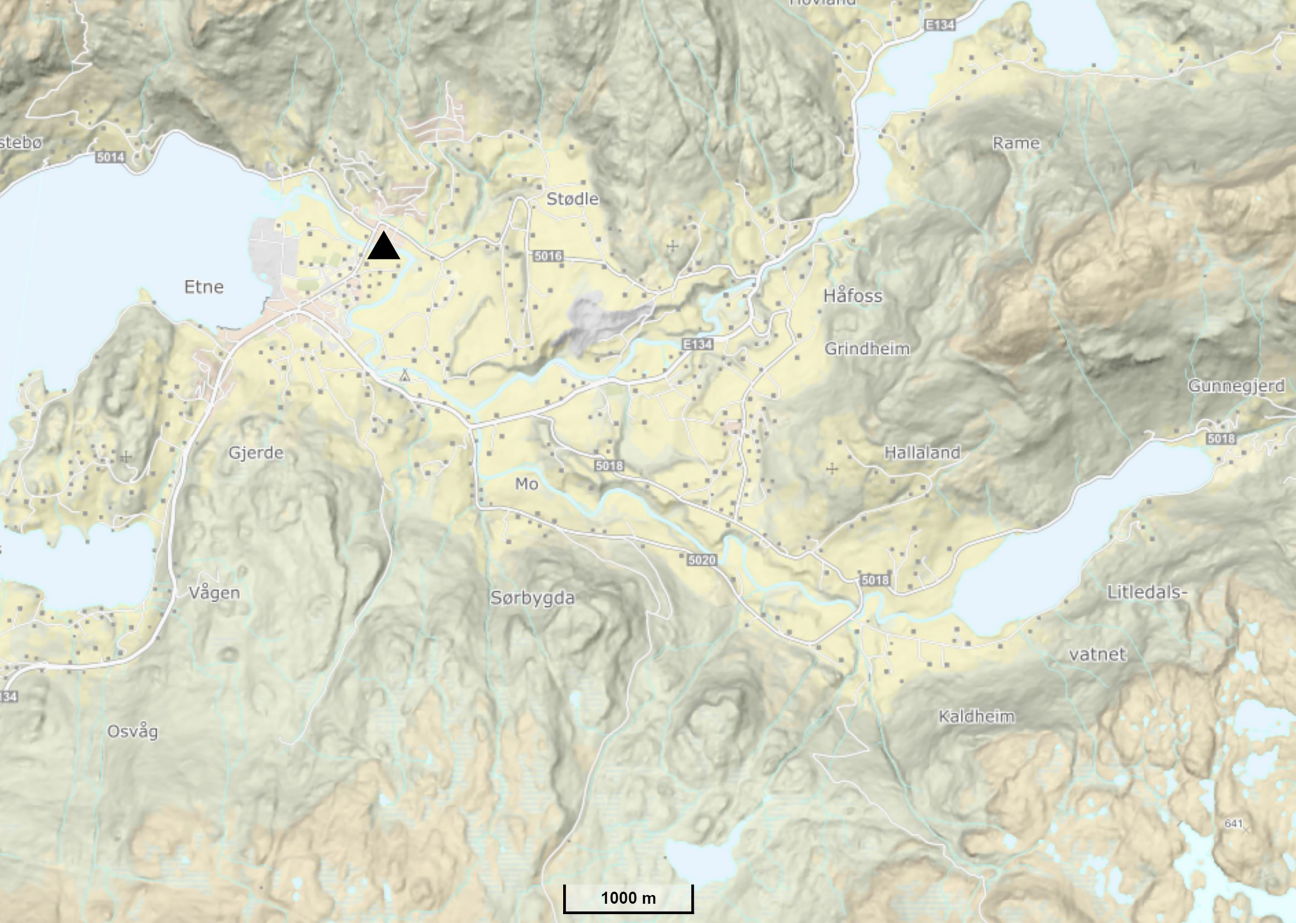
Location of the river Etne and the resistance board weir trap (black triangle) (Skaala et al., 2018). The map is retrieved from https://www.norgeskart.no/.

In 2013, an upstream fish trap was established in the river Etne, western Norway (Figure 1). This facility permits the recording and sampling of nearly all adult salmon entering the trap as they ascend the river. In the period 2013–2019, approximately 10,600 wild salmon were sampled upon entry to the river. All these fish were measured and then sampled for both tissue and scales thus permitting both molecular‐ and scale‐based identification of repeat spawners. The aims of our study were: (1) to identify and elucidate the proportions and patterns of growth and demography of repeat spawning in an Atlantic salmon population using complementary methods, (2) to examine the individual weight loss from time of river entry until leaving the river of out‐migrating kelts that were sampled while leaving the river in April and May 2018–2020 and (3) to briefly compare the frequency of repeat spawners identified by molecular methods versus scale analysis.

## 2 MATERIALS AND METHODS

### 2.1 Sampling

The river Etne, located in western Norway (Figure 1), has one of the major salmon populations in this region, with an annual angling catch ranging from 300 to 1000 fish during 1994–2019 (Lehmann et al., 2010; Skaala et al., 2015; www.etnelaks.no) and between 411 and 2164 salmon ascending the river from 2013 to 2019 (Harvey et al., 2022). The river has between 288,500 and 371,400 m^2^ of salmon‐rearing habitat, depending on water discharge (Hindar et al., 2007; Skoglund et al., 2008). The upstream migration trap was established in the river in 2013 to mitigate further potential impacts on the wild population from escaped domesticated salmon (Besnier et al., 2022; Glover et al., 2013) and permits sampling of most of the adult population. The trap is operated from April to November and catches both wild and escaped farmed salmon, allowing wild salmon to be released above the trap post‐sampling, while escaped farmed salmon are killed (Skaala et al., 2015, 2018).

The date of entry, weight (to the nearest 0.1 kg) and length (to the nearest centimetre from tip of nose to tip of caudal lobe) are recorded for all fish captured in the trap, and thereafter, 2–4 scales are sampled above the lateral line. The age at first maturity (hereafter called maiden sea age) and spawning status of the fish is determined by examining photographs of rinsed scales, and growth is determined using methodology described by Lea‐Dahl (Dahl, 1910; Lea, 1910). A small piece of tissue is removed from the adipose fin for DNA analysis (Skaala et al., 2015, 2018). Since the trap was installed in 2013, potential repeat spawners in 2013 and potential alternate repeat spawners in 2014 could not be identified by molecular methods alone, because they would have spawned for the first time in 2012 or earlier and accordingly, there were no available genotypes from that time that could be compared to later years. Therefore, only salmon entering the trap in the years 2015–2019 were used in the present study. Due to this, iteroparity between smolt year classes 2012–2016 could be compared for individuals identified by scale reading, as scales from fish entering the trap in 2013 would yield spawning information, while genetically identifying repeat spawners would only be possible for the smolt year classes 2014–2016. There were 8964 wild Atlantic salmon captured in the trap during the 2015–2019 period of which 8866 were scale read and it is these individuals and their genetic data (see below), together with scale reading of the same individuals, which form the basis of this study (Table 1).

**TABLE 1:** The number of maiden and repeat spawners by sex (a) and the number of repeat spawners by sex and spawning strategy (b) for the years 2015–2019 identified using genetics and the scale method: Scale RSP: those individuals identified as repeat spawners by the scale method only, Genetic RSP: those individuals identified as repeat spawners by the molecular method only, Both RSP: those individuals identified as repeat spawners by both methods. Note that total numbers may differ due to missing individual sex or spawning strategy information. Where F: female, M: male, A: alternate, C: consecutive. Also shown are the number of kelts by sex that were sampled in April–May in 2018–2020 (c). Total number of kelts each year may differ from the total of females and males due to some kelts not being identified back to themselves upon ascendance in previous years.

A	Female	Female	Female	Female	Male	Male	Male	Male
Year	Maiden	Scale RSP	Genetic RSP	Both RSP	Maiden	Scale RSP	Genetic RSP	Both RSP
2015	661	22	1	28	1365	20		7
2016	1138	33		17	850	31		19
2017	734	26	3	100	782	37	1	49
2018	607	24	2	79	697	7		27
2019	426	11	1	48	680	5		14

B	Female	Female	Female	Female	Female	Female	Male	Male	Male	Male	Male
	Alternative	Alternative	Alternative	Consecutive	Consecutive	Consecutive	Alternative	Alternative	Alternative	Consecutive	Consecutive
Year	Scale RSP	Genetic RSP	Both RSP	Scale RSP	Genetic RSP	Both RSP	Scale RSP	Genetic RSP	Both RSP	Scale RSP	Both RSP
2015	3	1	23	1		3	1		5	1	1
2016	2		8			8			3		15
2017		1	79	1	2	13	1	1	41		5
2018		2	71			6			21		6
2019			39			3			10		3

C	Female	Male
2018	37	10
2019	45	12
2020	38	3

In addition to the main data set consisting of fish captured in the trap, 171 (48 in 2018, 64 in 2019, 59 in 2020) kelts were caught on their outward river migration from April to May and represent a subsample of kelts leaving the river after spawning upstream. These kelts were captured by a mixture of angling by rod and line as well as using the upstream migration trap to herd downstream migrating fish into a fyke net. The same phenotypic measurements were taken for the kelts, including DNA samples, as for the upstream migrating adults.

### 2.2 Genotyping

All salmon were genotyped with 31 highly polymorphic microsatellite markers. The procedure of amplification and DNA extraction of 18 microsatellite markers (SSsp2201, SSsp2210, SSspG7, Ssa202, SsaD144, SsaD157, Sp1605, Sp2216, Ssa14, Ssa171, Ssa289, MHC1, MHC2, SSsp3016, SsOsl85, Ssa197, SsaD486 and SsaF43) followed the same laboratory protocol as in Harvey et al. (2017) and Quintela et al. (2016). The remaining 13 markers (EST107, EST19, EST28, EST68, Sleel53, Sleen82, SsOSL25, SsSP2215, Ssa405, Ssa407, Ssa412, Ssa98 and Ssleer15.1) were amplified in two multiplexes as in Harvey et al. (2017) and Ozerov et al. (2017). In addition, all individuals were genotyped to determine sex as in Harvey et al. (2017). PCR products were resolved on an ABI 3730 Genetic Analyzer and sized using a 500LIZ size standard (Applied Biosystems). Alleles were scored manually using Genemapper version 5.0. Independently of each other, two persons quality checked the scoring of microsatellite alleles before the data were exported to the database. Expected and observed heterozygosity, allelic richness and deviation from Hardy–Weinberg expectations were calculated using the *poppr* and *adegenet* packages in R (Jombart & Ahmed, 2011; Kamvar et al., 2015; Table S1 in File S2).

### 2.3 Identification of repeat spawners using the molecular method

The individual genotypes of all the fish sampled in the trap were compared within and between years to identify identical genotypes within the data set (i.e. to find a potential match back to itself in the database) and classify them in three ways: (1) repeat spawners were identified as individuals that were resampled in the trap in different years, (2) fish that displayed within‐river migration (i.e. moved up the river into the trap before moving back down the river over the trap and back up the river into the trap again) were identified as individuals that were resampled twice in the trap in the same year and (3) kelts were identified by matching the individual genotypes of the kelts sampled in the spring to the individual genotypes of fish sampled in the trap in the previous year.

Several programs exist to identify duplicate genetic data, however, the method used in the present study was developed in‐house to be able to have complete control over how missing genetic information or potential genotyping errors were handled in relation to the false discovery rate. A full description of the in‐house protocol used to identify repeat spawners using the molecular method is available in the File S2.

### 2.4 Identification of repeat spawners by scale analysis

The same individuals used in the genetic data set were also scale‐read to determine potential previous spawning history as mentioned above. Scale reading can also elucidate the spawning strategy (alternate vs. consecutive) of an individual. The analysis was carried out by trained personnel according to international guidelines for scale reading in Atlantic salmon (ICES, 2011). When an individual was identified as a repeat spawner using the molecular method but not identified using scales, the scales were re‐read to determine the reason behind the difference. However, the initial assessment of spawning status for each method was used in the analyses described below.

### 2.5 Statistical analysis

All statistics were conducted in R (version 4.2.2) (R Core Team, 2018). All analyses, unless stated otherwise, were performed on the repeat spawning individuals identified by both scales and the molecular method. The analyses pertaining to these individuals' spawning strategy were based on the number of years between maiden entry and entry as a repeat spawner as determined by the molecular method, and individuals who had more than 1 year between spawning events (*N* = 4) were grouped within the alternate category. Sea age at first maturity (1SW: one sea winter, 2SW: two sea winter, MSW: multiple sea winter) was based on the sea age determined by scale reading for maiden spawners.

Chi‐square tests were used to investigate the overall differences in the proportion of repeat spawners identified by the molecular vs scale method, including between the sexes and strategies. Chi‐square tests were used to investigate whether the proportion of iteroparity changed over the years within smolt year classes 2012–2016 based on repeat spawners identified by scales and smolt year classes 2014–2016 identified by the molecular method. Finally, chi‐square tests were used to analyse whether the distribution of sea age at first maturity differed between maiden and future repeat spawners over the years 2015–2018.

#### 2.5.1 Investigating the demography of repeat spawners

The demography of the repeat spawners identified by both methods was examined with a generalized linear model (glm) where the number of repeat spawners was predicted as the response to sex (2 levels: female and male), maiden sea age [3 levels: 1 sea winter (SW), 2SW and multiple SW], spawning strategy (2 levels: alternate and consecutive), year (5 levels: 2015–2019) and the two‐way interactions of sex and strategy, sex and maiden sea age, strategy and maiden sea age, maiden sea age and year.

(1.1) $$\begin{array}{c} Y=\mathrm{Sex}+\mathrm{Sea}\;\mathrm{age}+\text{Strategy}+\text{Year}+\mathrm{Sex}\times \mathrm{Sea}\;\mathrm{age}+\mathrm{Sex}\\ \;\times \text{Strategy}+\mathrm{Sea}\;\mathrm{age}\times \text{Strategy}+\mathrm{Sea}\;\mathrm{age}\times \text{Year}, \end{array}$$

where *Y* is the number of repeat spawners observed, modelled using a negative binomial distribution with a log‐link function. It was not possible to include a two‐way interaction between strategy and year due to model convergence issues. Analysis of variance (ANOVA) with a type II chi‐square test was used to assess the significance of the predictors. Model fit was examined using the DHARMa package (Hartig, 2022). For significant two‐way interactions, pairwise comparisons between each level of the variable were conducted using the pairs function from the emmeans (estimated marginal means) package (Lenth, 2019) with the default Tukey adjustment for multiple comparisons.

The gain in growth observed between maiden and second river entries for repeat spawners was modelled as a linear response to sex (2 levels: female and male), spawning strategy (2 levels: alternate and consecutive), maiden sea age [3 levels: 1 sea winter (SW), 2SW, and multiple SW] and the maiden year of ascendance (2013–2017) together with pairwise interactions between the predictors. The full model was as follows:

(1.2) $$\begin{array}{c} Y=\mathrm{Sex}+\mathrm{Sea}\;\mathrm{age}+\text{Strategy}+\text{Maiden year}+\mathrm{Sex}\times \mathrm{Sea}\;\mathrm{age}\\ \;+\mathrm{Sex}\times \text{Strategy}+\mathrm{Sea}\;\mathrm{age}\times \text{Strategy}+\mathrm{Sea}\;\mathrm{age}\\ \;\times \text{Maiden year}+\mathrm{Sex}\times \text{Maiden year}, \end{array}$$

where *Y* is the change in length from the first registration in the trap as a maiden spawner and the second registration in the trap at repeat spawning modelled using a Gaussian distribution. All explanatory variables were as above in model 1.1 apart from the maiden year (year of maiden entry to the river) being used here, which consisted of five levels due to low numbers in 2018 (2013–2017). Model selection was conducted by comparing AIC values (second‐order Akaike Information Criterion) for models containing subsets of the fixed variables in the full model, where the best‐fitting model displayed the lowest AIC value. The significance of the fixed variables was then assessed by an ANOVA, and post hoc Tukey HSD tests compared the significance of differences between the groups within a significant fixed variable as above. Model fit was assessed as above. The total weight per year of the female repeat spawners was used to calculate an approximate estimate of their fecundity according to Hindar et al. (2007), where egg deposition is calculated as 1450 eggs/kg body mass.

Generalized linear models were used to investigate the relationship between the date when entering the river for a second time to spawn again (hereafter referred to as second river entry, i.e. repeat spawning) and the date at the first river entry (maiden), and whether this differed between the sexes, maiden sea age or maiden years:

(1.3) $$\begin{array}{c} Y=\text{Maiden}\;\mathrm{day}\;\text{of arrival}+\mathrm{Sex}+\text{Maiden}\;\mathrm{sea}\;\mathrm{age}+\text{Maiden year}\\ \;+\mathrm{Sex}\times \text{Maiden}\;\mathrm{sea}\;\mathrm{age}+\mathrm{Sex}\times \text{Maiden year}\\ \;+\text{Maiden}\;\mathrm{sea}\;\mathrm{age}\times \text{Maiden year}, \end{array}$$

where Y is the day of the year at second river entry (repeat spawning) modelled using a negative binomial distribution with a log link. Due to modelling constraints, it was decided to analyse strategies (alternate or consecutive) separately. Due to the lower number of consecutive repeat spawners, there were no interactions included in that model.

Model selection and the post‐hoc comparisons of significant terms were conducted as above. The observations of the third‐time spawners were not included in the analyses due to low numbers.

#### 2.5.2 Investigating the proportion of male and female kelts

The differences in the proportion of male and female kelts within the years 2017–2019 were investigated with two sample chi‐square tests. A linear model was used to investigate the relationship between weight loss and time spent in the river for the captured kelts.

(1.4) $$\begin{array}{c} Y=\text{Time in the river}+\text{Ascendance year}+\mathrm{Sex}+\mathrm{Sex}\\ \;\times \text{Ascendance year}+\text{Time in the river}\times \mathrm{Sex}\\ \;+\text{Time in the river}\times \text{Ascendance year}, \end{array}$$

where Y is the weight difference (kg) between weight at ascending the river and weight as a kelt leaving the river the following year. Model selection and the post hoc comparisons of significant terms were conducted as above.

## 3 RESULTS

### 3.1 Incidence of repeat spawning

A total of 405 repeat spawning events were identified in the entire data set (*N* = 8866) by matching individual genetic profiles among different years (2015–2019) (Table 1). This consisted of 378 unique individuals that had entered the river two or more times to spawn in the 2015–2019 period. Most of the repeat spawners were second‐time spawners, however, 25 individuals were third‐time spawners, and a single female was recorded in five separate consecutive years in the trap. The overall frequency of repeat spawners detected by molecular methods in the adult population in the period 2015–2019 was therefore estimated to be 5% (2%–8% between the years). A total of 617 repeat spawning events were identified using the scale method, with an overall frequency of 7% (4%–12% between the years) detected between 2015 and 2019. Of the entire spawning population, the scale method identified significantly more female (average 10%, 4%–14%) and male repeat spawners (average 5%, 2%–10%) than the molecular method (females average 7%, 1%–12% and males 3%, 1%–6%). Both methods identified a similar frequency of repeat spawners that were 1SW at the age of first maturity (scale: 3% vs molecular: 3%) and 2SW at first maturity (scale: 7% vs molecular: 6%), while the scale method identified significantly more MSW repeat spawners (22%) than the molecular method (6%). There were significantly fewer alternate strategy repeat spawners identified by scales compared to the molecular method (74% vs 84%), but significantly more consecutive strategy spawners identified by scales compared to the molecular method (26% vs 16%). The frequency of iteroparity varied significantly between smolt year classes for repeat spawners identified by both scales (smolt year classes 2012–2016, 2%–31% iteroparity, *p*‐value < 0.001) and the molecular method (smolt year classes 2014–2016, 1%–6%, *p*‐value < 0.001), with no clear trend among the years. Using only those individuals in agreement among the methods, within 2015–2017, there were significantly higher proportions of repeat spawners whose sea age at first maturity was 2SW compared to maiden spawners maturing as 2SW fish. See Table S1 for all comparisons and *p* values.

The methods congruently identified 386 repeat spawning events in the data set (5%). Of these, there were 364 unique spawners who spawned more than once in the river. There were 19 individuals who were identified as repeat spawners by the molecular method but not by scale reading. The scales from these individuals were re‐read by the same individual, and results from the second reading showed 5 non‐readable scales, 12 individuals where the spawning mark was weak and not initially spotted and 2 individuals with no scale sample.

**FIGURE 2:**
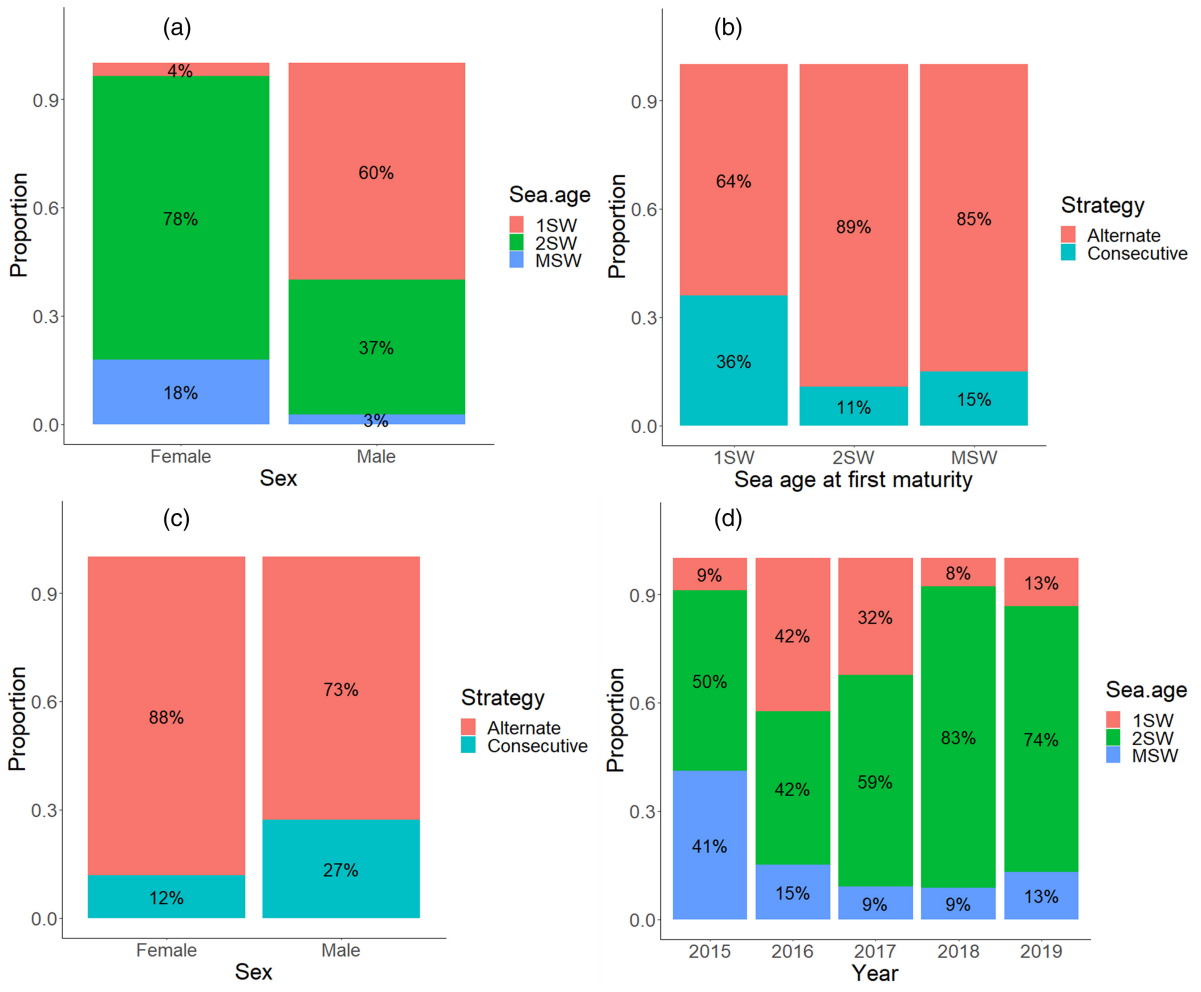
The proportion of repeat spawners identified by both scales and the molecular method by (a) sea age at first maturity within the sexes, (b) spawning strategy within the sea age at first maturation, (c) spawning strategy within the sexes and (d) sea age at first maturity within each year. Percentages of each group are presented within the graphs.

The ANOVA output for the model 1.1 is presented in Table 2a. Post‐hoc multiple comparisons are presented in Table S2. Overall, there were significantly more female (70%) than male (30%) repeat spawners, and the within‐year differences were not significant. Most repeat spawners had matured for the first time as 2SW fish (66%), and there was no difference in the frequency of repeat spawners who first matured as 1SW (21%) or MSW fish (13%). The frequencies of the sea ages at first maturity among the sexes of the repeat spawners were all significantly different (Figure 2a). Most female repeat spawners first matured as 2SW fish (78%), followed by first maturation as MSW fish (18%), while most of the males had matured for the first time as 1SW fish (60%) followed by first maturation as 2SW fish (37%). There were significantly more alternate (83%) than consecutive (17%) repeat spawners overall, and although the proportion of male consecutive repeat spawners (27%) was higher than female consecutive repeat spawners (12%), the differences in distributions of repeat spawners between these two strategies did not differ significantly between the sexes (Figure 2c). There were consistently more alternate than consecutive repeat spawners for all sea ages at first maturity, although after correction for multiple comparisons only the differences between strategies for 2SW fish (89% vs 11%) and MSW fish (85% vs 15%) were significant (Figure 2b). The frequency of repeat spawners varied across the years, with the highest number in 2017 (37% of all repeat spawners entered the trap in 2017) and the lowest in 2016 (9% of all repeat spawners), with significant differences between 2015 and 2017 and 2016 and between 2016 and 2019. As mentioned previously, most repeat spawners had matured as 2SW fish, and this trend was evident in all years apart from 2016 when the frequencies of repeat spawners first maturing as 1SW and 2SW fish were equal (Figure 2d). Differences between the frequencies of the sea age at first maturity of the repeat spawners within the years were significant when comparing MSW fish (9%) to 1SW (32%) and 2SW fish (59%) in 2017, the 2SW fish (83%) compared to 1SW (8%) and MSW fish (9%) in 2018 and between 2SW (74%) and MSW (13%) fish in 2019 (Figure 2d).

**TABLE 2:** ANOVA output of the generalized linear models and linear models investigating (a) the proportion of repeat spawners, (b) the change in length of the repeat spawners and the date of second entry of alternate strategy (c) and consecutive strategy (d) repeat spawners.

	Model terms	Chi square	Df	*p* value
A Model 1.1	**Sex**	**13.29**	**1**	**0.000**
	**Sea age**	**28.16**	**2**	**0.000**
	**Strategy**	**47.62**	**1**	**0.000**
	**Year**	**30.48**	**4**	**0.000**
	Sex × Strategy	0.30	1	0.584
	**Sex × Sea age**	**54.04**	**2**	**0.000**
	**Sea age × Strategy**	**8.89**	**2**	**0.012**
	Sex × Year	3.44	4	0.487
	**Sea age × Year**	**21.72**	**8**	**0.005**
B Model 1.2	**Strategy**	**347.33**	**1**	**0.000**
	Sex	0.18	1	0.674
	**Sea age**	**208.91**	**2**	**0.000**
	**Maiden year**	**34.62**	**4**	**0.000**
	Sex × Maiden year	9.76	4	0.045
	Sea age × Maiden year	9.43	8	0.307
	Sex × Sea age	2.22	2	0.330
	Sex × Strategy	0.50	1	0.478
	**Sea age × Strategy**	**12.75**	**2**	**0.002**
C Model 1.3a	**Maiden day of arrival**	**73.41**	**1**	**0.000**
	**Sex**	**6.46**	**1**	**0.011**
	Maiden sea age	0.89	2	0.640
	Maiden year	58.82	4	0.000
	Sex × Maiden sea age	0.62	2	0.734
	Sex × Maiden year	1.34	4	0.855
	Maiden sea age × Maiden year	3.97	8	0.860
D Model 1.3b	**Maiden day of arrival**	**6.64**	**1**	**0.010**
	Sex	0.17	1	0.681
	Maiden sea age	0.56	2	0.757
	Maiden year	9.69	4	0.046

*Note*: Significant terms are shown in bold. Df: degrees of freedom.

**FIGURE 3:**
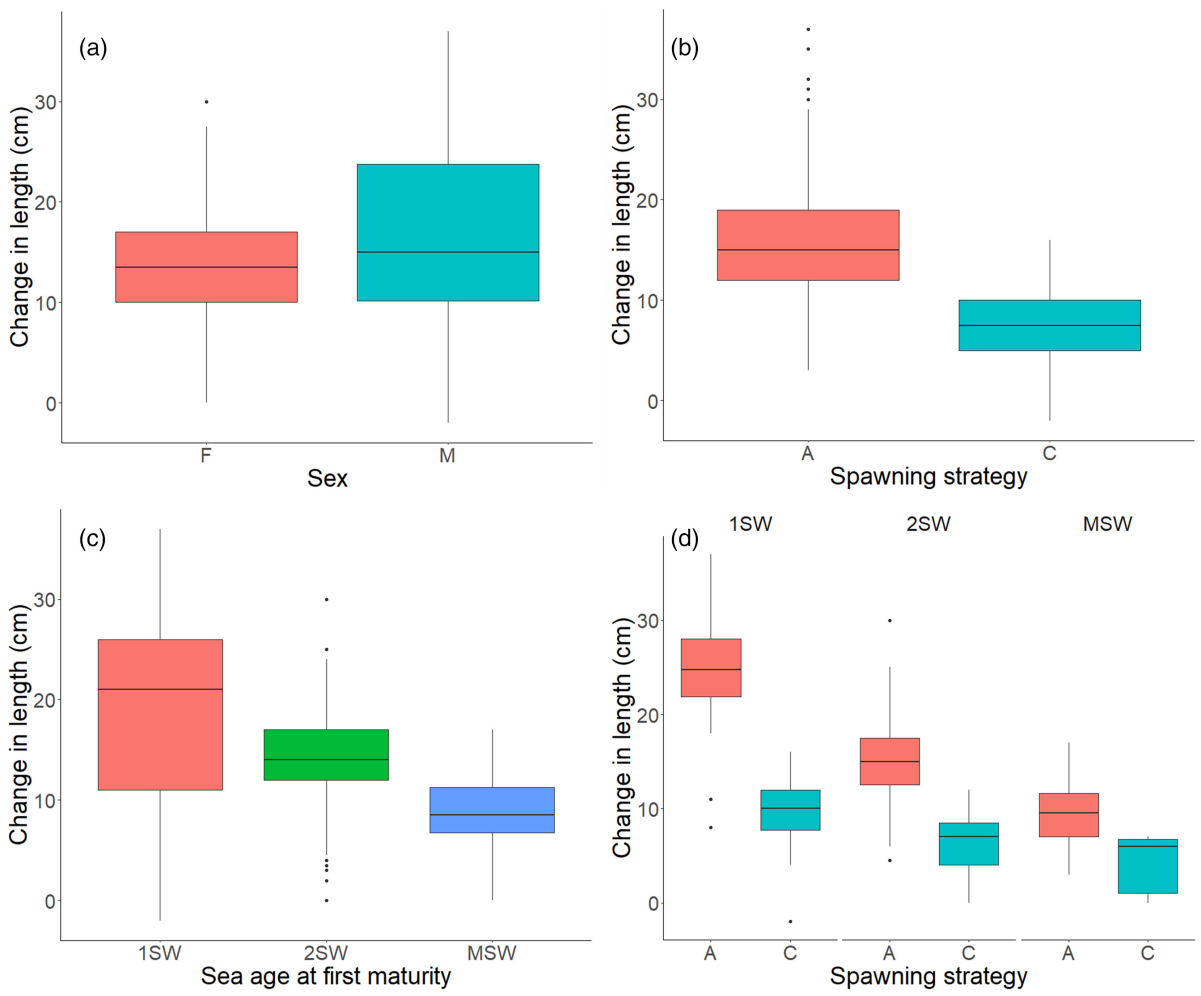
The average length (cm) difference from maiden river entry to entry as a second‐time repeat spawner identified by both methods for (a) females and males, (b) alternate and consecutive strategies, (c) sea age at first maturity and (d) spawning strategy within sea ages at first maturity. The median value is shown as the black line inside the box, whereas the lower and upper lines defining each box is the lower and upper quartiles. Outliers are shown as black points.

The ANOVA output for model 1.2 is presented in Table 2b. Post‐hoc multiple comparisons are presented in Table S3. Repeat spawners gained an average of 14.4 cm in length from maiden entry until entry at second spawning (Figure 3). There was no significant difference in the average change in length from maiden spawning to repeat spawning between the sexes. Alternate repeat spawners gained on average 15.9 cm in length compared to consecutive repeat spawners (7.7 cm gained) (Figure 3b) and repeat spawners that first matured as 1SW fish gained significantly more than those who matured first as 2SW fish and MSW fish (19.9 cm vs 14.1 cm and 8.9 cm, respectively) (Figure 3c). Comparing the size gained for the different spawning strategies of the fish maturing at the same age, alternate repeat spawners gained significantly more in length (1SW: 24.8 cm, 2SW: 15.0 cm, MSW: 9.7 cm) than consecutive repeat spawners (1SW: 9.9 cm, 2SW: 6.4 cm, MSW: 3.6 cm) (Figure 3d). The average change in length from maiden spawning to repeat spawning differed among maiden years of spawning (range 11.6–16.7 cm over the years), however, these differences were not significant after correction for multiple comparisons.

The total weight of first‐time spawning females captured in the trap in the years between 2015 and 2019 was 2518, 4493, 3514, 2785 and 2007 kg, respectively. The total weight of the female repeat spawners identified by both methods for the same years was 183, 115, 661, 582 and 355 kg. Using the approximate egg calculation (Hindar et al., 2007) of 1450 eggs/kg body mass, this equates to an estimated potential deposition of 2,747,286 eggs from repeat spawners during the period 2015–2019, or put alternatively, an estimated potential spawning contribution in the entire population of 7%, 3%, 19%, 21% and 18% per year respectively (average = 12%).

**FIGURE 4:**
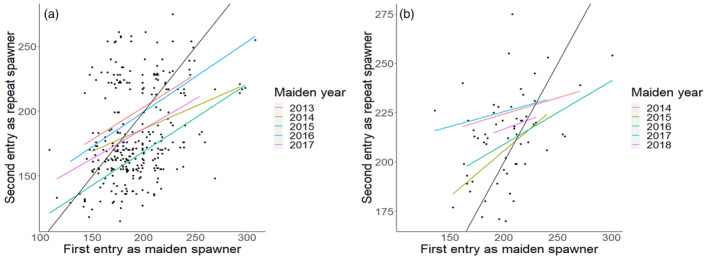
The day of the year that repeat spawners identified by both methods ascended the river Etne for the first time as maiden spawners and as (a) alternate strategy and (b) consecutive strategy repeat spawners. Maiden year is the year of entry to the river as a first time spawner. The black line represents the line through the origin.

The ANOVA outputs for the models described in 1.3 are presented in Table 2c,d. Post‐hoc multiple comparisons are presented in Table S4. The date of second entry as a repeat spawner was positively and moderately correlated with the date of entry as a maiden spawner for both alternate and consecutive repeat spawners (Figure 4) demonstrating that individuals entering the river early or late in the season as maidens tended to keep a similar timing as repeat spawners. Although not directly compared, consecutive repeat spawners entered the river on average 20 days later than alternate repeat spawners. For both strategies, individual fish entered the river later the second time than the first time (average 11 days for alternate and 10 days for consecutive).

For alternate repeat spawners, the second date of entry for females was on average 8 days later than males, and the average date of second entry to the river varied among the years (range: day 171–day 197), although after correction for multiple comparisons, only the difference between 2015 and 2016 for alternate repeat spawners was significant (19 days). For consecutive repeat spawners, there was no significant difference in day of river entry between the sexes, and the average date of second entry varied among the years from day 209 to day 225.

### 3.2 Kelts

A sample of 48, 64 and 59 kelts were captured on their seaward migration during the spring of 2018, 2019 and 2020 respectively (Table 1). Of the kelts sampled, 47 (98%), 57 (89%) and 41 (69%) were identified back to themselves upon ascendance in 2017, 2018 and 2019, respectively, and 24 of these had been identified as previous repeat spawners. Kelts that were not matched back to themselves at ascendance were excluded from further analysis.

In 2017, a total of 880 females and 878 males were captured and sampled on ascending the river, and 37 (4%) and 10 (1%) of these were captured as kelts in the following spring. In 2018, 733 females and 748 males were captured and sampled on ascending the river and 45 (6%) females and 12 (2%) males were captured as kelts. In 2019, 488 females and 699 males were captured and sampled on ascending the river. Of these, 38 (8%) females and 3 (0.4%) males, respectively, were captured as kelts. There were significantly more female kelts captured during the period than males in all years (2017: 79% vs 21%, 2018: 79% vs 21%, 2019: 93% vs 7%) (Table S5).

**FIGURE 5:**
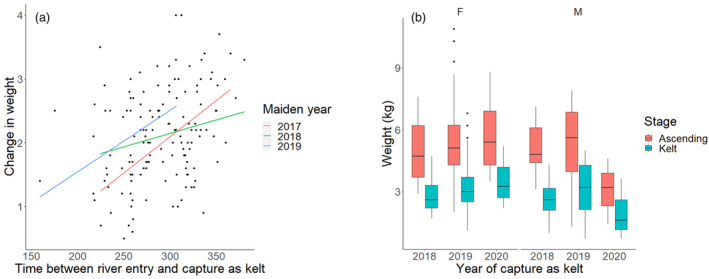
The (a) change in weight from entry to the river as a spawner until capture as kelt over the time elapsed in the river. Maiden year is the year of entry to the river. (b) The average weight as an ascending spawner and kelt for females (F) and males (M) within each year of capture as a kelt.

The ANOVA output for model 1.4 is presented in Table 3. The relationship between the change in weight from spawner to kelt and the time elapsed from entry to the river until capture as a kelt was significant and positive, indicating that weight loss increased with time spent in the river (Figure 5a). The average decrease in weight among the kelts was 2.1 kg, an average of 40% of their body weight lost while in the river, which equates to an average loss of 7 grams of body weight per day.

**TABLE 3:** ANOVA output of the generalized linear model investigating the change in weight of the kelts captured in 2018–2020.

	Model terms	Chi square	Df	*p* value
Model 1.4	**Time elapsed**	**21.99**	**1**	**0.00**
	Maiden year	3.56	2	0.17
	Sex	2.18	1	0.14
	Sex × Maiden year	2.42	2	0.30
	Sex × Time elapsed	1.93	1	0.17
	Time elapsed × Maiden year	4.92	2	0.09

*Note*: Significant terms are shown in bold. Df: degrees of freedom.

## 4 DISCUSSION

Using the phenotypic and genetic profiles for >8000 wild Atlantic salmon entering the trapping facility located in the river Etne in the period 2015–2019, in addition to sampling kelts over 3 years, this study has provided insights into the proportions and biological patterns of repeat spawning regarding size changes over time, differences among sexes and spawning strategies, and within sea age at first maturation. Furthermore, this is the first study to compare the classification of repeat spawners in Atlantic salmon using genetic and scale analysis. The main results can be summarized as follows: the frequency of repeat spawners in this period detected by both methods congruently was 7% in females and 3% in males, most of the repeat spawners (88% females, 73% males) spent one whole year reconditioning at sea before returning for their second spawning, with a substantially larger body size compared to their maiden size. There was a correlation between the ascendance timing in maiden and second river entry years of the repeat spawners and consecutive repeat spawners often entered the river later than alternate spawners. Between maiden river entry and second river entry, repeat spawners gained an average of 2.5 kg of body weight and grew an average of 14.2 cm, and weight gain was dependent on the repeat spawning strategy. Repeat spawners contributed an average of 12% of the potential egg production in the river in the period 2015–2019, with contributions as high as 21% in a single year. On average, kelts lost 40% of their body weight, which equated to ~7 g of body weight per day in the river, and more female than male kelts were captured during outward migration in April and May. Identification of repeat spawners using scales vs the molecular method revealed that the molecular method tended to underestimate the frequency of iteroparity, however, trends across years and sexes were similar between the approaches. Genetic identification permitted unique insights into patterns of repeat spawning in individual fish such as individual information on the repeat spawners including size differences and timing of river entry between spawning events.

### 4.1 Iteroparity in Atlantic salmon

The overall frequency of repeat spawners in the river Etne estimated over the period 2015–2019 identified by both methods was 5%. Most (94%) of the repeat spawners were second‐time spawners, but 25 fish returning to spawn for a third time were also recorded. Remarkably, a single female was recorded partaking in her fifth spawning migration. Repeat spawners who have spawned multiple times have been documented previously in adult Atlantic salmon (Bøe et al., 2022; Fleming, 1996; Halttunen, 2011; Jonsson, Hansen, & Jonsson, 1991; Mills, 1989; Niemelä, Erkinaro, et al., 2006), and Ducharme (1969) identified individual salmon that had spawned four and five times, respectively. Scale readings of a 14‐year‐old female that had spawned on four occasions were reported in the river Tana (Erkinaro et al., 2019).

The frequency of repeat spawners identified in the present study is comparable to the proportions of repeat spawners observed in other rivers in Norway, Iceland, Ireland, and North America (Bordeleau et al., 2020; Erkinaro et al., 2019; Jonsson & Jonsson, 2004; Kjartansdottir, 2008; Persson et al., 2022). The large energetic cost of the spawning migration, spawning itself and overwintering, along with the high marine mortality of kelts exiting the river, may explain why most adult salmon only spawn once (Jonsson, Hansen, & Jonsson, 1991; Jonsson & Jonsson, 2004; Jonsson, Jonsson, & Hansen, 1991; Klemetsen et al., 2003). A higher proportion of repeat spawning females than males, as observed in this study, has also been reported in earlier studies (Bordeleau et al., 2020; Halttunen, 2011; Harvey et al., 2022; Kjartansdottir, 2008; Mills, 1989; Persson et al., 2022). The energetic cost of reproduction differs between the sexes in Atlantic salmon, with males less likely to be iteroparous (Bøe et al., 2022). Males display aggressive behaviours towards each other prior to and during spawning, which may lead to injuries and higher mortality compared to females, who allocate most of their energy to egg production (Fleming, 1996; Halttunen, 2011; Jonsson, Jonsson, & Hansen, 1991; Niemelä, Erkinaro, et al., 2006).

We observed that most repeat spawners displayed an alternate strategy, consistent with observations from other studies in other Norwegian rivers (Persson et al., 2022), including the rivers Tana (Erkinaro et al., 2019; Niemelä, Erkinaro, et al., 2006) and Alta (Halttunen, 2011). Repeat spawners that displayed an alternate strategy gained more weight than consecutive spawners. Both Jonsson, Hansen, and Jonsson (1991) and Bøe et al. (2022) have suggested that repeat spawning strategies are related to body size, whereby the proportion of alternate repeat spawners increases with size and age at first spawning. However, the repeat spawning strategy may not depend on size alone, as we observed differences between the sexes, with males displaying a consecutive strategy more often than females, although this was not a significant difference in our study. Reid and Chaput (2012) observed a smaller egg size in consecutive females than alternate females, suggesting that females may spend more time reconditioning at sea to increase their reproductive success and offspring fitness through both increased fecundity and egg size. Bøe et al. (2020) found differences in the fatty acid profiles that were linked to different oceanic feeding grounds between repeat spawning strategies, indicating that alternate and consecutive repeat spawners may display different migratory behaviours. These differences may carry over to affect various traits such as body condition, egg quality and even survival (Bøe et al., 2020).

Temporal variation in the proportion of repeat spawners has been observed in the river Tana in northern Europe. Niemelä, Erkinaro, et al. (2006) examined the variation in the proportion of repeat spawning anadromous salmon, based on the analysis of fish scales sampled over a 30‐year (1975–2004) period and found that the proportion ranged from 1% to 29% for females and <1%–14% for males. Inter‐annual variation in the proportion of repeat spawners has been observed in other salmon populations in the northwest Atlantic (Bordeleau et al., 2020). In the current study, the yearly frequency of repeat spawning females and males in the population varied between 1% and 11% and between 1% and 6% respectively. A study on the same river found that the frequency of repeat spawners identified by scales had increased over time by comparing historical samples (3%, 1983 and 1984) to contemporary samples (7%, 2017 and 2018) (Harvey et al., 2022). As most of the female repeat spawners displayed an alternate strategy, the very low proportion of female repeat spawners observed in the river Etne in 2016 is likely a consequence of the extremely low abundance of spawners entering the river in 2014 (Table 1). The proportion of repeat spawning males in 2016 was not lower than in 2015 or 2019. This is in accordance with males displaying a consecutive strategy more often than females, and the very high number of first‐time spawning males in 2015 (Table 1). It is worth noting that in 2014, when a low number of spawners was recorded entering the river (*N* = 411), 75 of these fish were identified as repeat spawners by scale analysis, that is, 18% of the spawning population were previous spawners (Harvey et al., 2022).

The timing of ascendance into a river is strongly influenced by environmental factors such as temperature, river discharge and water levels (Dahl et al., 2004; Harvey et al., 2017; Jonsson et al., 2007). In addition, timing of river entry is also influenced by biological factors such as sex, size and age, as larger and older multi‐sea winter fish generally ascend the river earlier than smaller one sea winter fish (Borgstrøm et al., 2010; Harvey et al., 2017; Thorstad et al., 2010), and females have been found to enter the river earlier than males (Dahl et al., 2004; Davidsen et al., 2013; Harvey et al., 2017). Among repeat spawners, Niemelä, Orell, et al. (2006) compared one, two and three sea winter first‐time spawners with repeat spawners that were one, two and three sea winters at first spawning and had spent one full year feeding at sea before the second spawning. They observed, from scale analysis and the registered time of capture by different fishing gears, that one sea winter alternate repeat spawners were captured earlier than one sea winter maiden spawner, and two sea winter alternate repeat spawner females were captured earlier than two sea winter females. Shearer (1990), on the other hand, found using scale analysis and registered time of capture from both angling and a trapping facility, that repeat spawners returned for their repeat spawning approximately at the same time as they did as maidens. For example, a repeat spawner which migrated to the river for the first time early in the season would also return to the river for their second spawning early in the season (Shearer, 1990). In the present study, we found a positive but moderate correlation between timing of first and second entry to the river for individual repeat spawners identified by both methods (Figure 3). Collectively, these observations may suggest a genetic component to the timing of river entry (Birnie‐Gauvin et al., 2021), which has also been shown in previous studies investigating the run timing of maiden spawners in both Atlantic salmon and sea trout (Cauwelier et al., 2017; Hansen & Jonsson, 1991; Stewart et al., 2002).

### 4.2 Characteristics of kelts

Among the 145 kelts sampled between April and May that were matched to spawners, there was a higher proportion of females than males in all 3 years. Our observations could suggest a higher survival of females post‐spawning, at least at this stage of migration, which is supported by the lower proportion of observed male kelts compared to the proportion of observed male repeat spawners. However, the lower proportion of male kelts may also be explained by their earlier out‐migration compared to female kelts, which in turn could explain why there is a larger proportion of male consecutive repeat spawners compared to females, as they would have more time in the ocean to recondition before returning to spawn. However, our sampling period for kelts was not exhaustive, and it was not possible for us to quantitatively disentangle post‐spawning within‐river mortality versus differences in out‐migration timing for the observed differences in the numbers of each sex captured.

Anadromous salmon do not normally feed when they return to freshwater to spawn but instead rely on their energy reserves gained during the sea migration to maintain body functions (Johansen et al., 2010). During river migration and spawning, salmon lose much of their body reserves, and kelts that overwinter in the river have poor body condition (Halttunen et al., 2010, 2013; Jonsson et al., 1997; Niemelä et al., 2000). In this study, the weight of the kelts leaving the river in the spring was substantially lower than upon ascendance (Figure 5b). The weight loss during winter was positively correlated with the number of days spent in the river (Figure 5a), which indicates a day‐by‐day weight loss, in addition to the expenditure of gametes during spawning itself. Jonsson et al. (1997) examined the energy expenditure in salmon related to upstream migration and spawning in the river Drammen and found that the total energy loss of migration and spawning was higher for large than for small individuals.

### 4.3 Comparison of scale and genetic analysis

The proportion of repeat spawners identified using scale analysis was consistently, although marginally, higher than the proportion identified by genetics (7% vs 5% in total). However, the patterns of repeat spawners within and between sexes and years were similar when using either method. The largest difference in the proportions estimated by the methods was observed when classifying the spawning strategies over the years. Scale analysis identified a higher proportion of the consecutive strategy among the repeat spawners than the molecular method (26% vs 16% in total). Studies that compare the use of scale analysis and genetic analysis to identify repeat spawning are rare, and to our knowledge this is the first comparison using Atlantic salmon. Seamons et al. (2009) used 12 microsatellite loci and scale readings to classify repeat spawners and age in steelhead trout in two rivers in Washington State, USA. They found, similar to our results, that scale analysis identified more repeat spawners than molecular methods. Of those fish identified as repeat spawners by genetics in that study, only two individuals were not also identified using scale analysis (referred to as a 6.5% error rate) (Seamons et al., 2009). The authors could only speculate that the fish identified by scales but not by genetics had either not been sampled previously (jumped the weirs) or that genotyping errors prevented genetic identification, although they concluded that genotyping error was not likely to be a cause. They also concluded that this implies but does not demonstrate that scale analysis may incorrectly identify some fish as repeat spawners (Seamons et al., 2009). Our results agree with those of Seamons et al. (2009) in that the direction of error in estimating repeat spawners is skewed towards underestimation using molecular methods. Whether this indicates an error in scale analysis or genetic analysis is not easy to conclude. Scale analysis may underestimate the proportion of repeat spawners due to scale degradation or overestimate the proportion of repeat spawners due to human error in the scale reading process. In the present study, of those fish that were not identified as repeat spawners by the scale method and were re‐read, over half had very weak spawning marks which were not flagged by the reader. Conversely, genetics may underestimate the number of repeat spawners due to a lack of a genetic sample which could be attributed to a fish jumping the trap, spawning in the short section of river below the trap, or previously having spawned in another river. In the present study, only 89% and 69% of the kelts sampled in 2019 and 2020 could be matched back to themselves ascending the trap in the previous season, indicating that some level of underreporting of repeat spawners may be expected when using genetic methods. In periods with high water levels, the catch efficiency of the trap is slightly reduced, and some fish may pass undetected. Jonsson et al. (2003) investigated straying from the river Imsa among tagged hatchery‐reared and wild post‐smolts returning as adults. Based upon recaptures of salmon in other rivers, straying of wild salmon was estimated as 6%. Studies investigating straying among Atlantic salmon have observed straying rates ranging from 0 to 10% (Altukhov & Salmenkova, 1994; Keefer & Caudill, 2014). However, post‐spawners transplanted to another river from the first spawning were unable to learn the new migration route and returned for repeat spawning in the same river as at first spawning (Hansen & Jonsson, 1994). This suggests straying may be less common among repeat spawners than maiden spawners, although, more research is required to estimate the straying rate of repeat spawners.

Using genetic data to estimate frequencies of repeat spawners is arguably a more precise method than scale analysis and can offer unique insights at the individual level on migration timing and growth over time. Nevertheless, only a low number of salmon rivers have traps which allow such detailed sampling as in the river Etne, and accumulation of such large genetic data sets covering multiple years is resource demanding and not possible under most circumstances.

## 5 MANAGEMENT IMPLICATIONS

Knowledge of the frequency and maintenance of iteroparity in salmonids should be a priority for the management of sustainable population levels and exploitation rates. Understanding the energetic dynamics of spent salmon spawners and the migration trends of returning salmon is vital to be able to adequately monitor and predict current and future challenges that Atlantic salmon may face. The spawning contribution of repeat spawners in the present study indicates that repeat spawners may contribute significantly to population recruitment, particularly in years with poor return rates of maiden spawners through buffering with reproductive contributions from more than just 1‐year class. Similarly, iteroparity can also be a significant source of genetic variability. River managers should take repeat spawners into consideration when setting exploitation rates and monitoring the status of river populations, particularly for recovering populations where repeat spawners may be important for population resilience and recovery.

## CONFLICT OF INTEREST STATEMENT

The authors declare no conflict of interest.

## Supporting information


File S1.

File S2.

## Data Availability

Data used in this article are available as a supplementary file.
