# Supplementary material for: DNA and scale reading to identify repeat spawning in Atlantic salmon: Unique insights into patterns of iteroparity
Source: Evol Appl. 2023 Nov 23;16(12):1921–36. doi: 10.1111/eva.13612 (PMC10739089; doi:10.1111/eva.13612)
Supplement: Supplementary file 2 — File S2. [file EVA-16-1921-s002.docx]

*Description of the molecular method*

Ideally, the same individual represented twice in a dataset would display an identical genotype at all 31 loci. However, missing genotypes and potential genotyping errors, as exist in low frequencies in almost all comprehensive DNA datasets, may reduce the number of exact matches (Suppl. Figure 1). Therefore, several loci may not be scored (i.e. missing) in one of the samples of a given individual and potentially one or two genotyping errors also exist in one or both of the DNA isolates that are being compared. In addition, two different individuals may display identical genotypes at a number of loci merely by chance. This chance match increases with a lower number of loci (Glover et al. 2012. Therefore, the decision to classify two identical genotypes as the same individual represented twice in a dataset is associated with a risk of a false positive match. Given these two sources of potential error, it was therefore necessary to first estimate the probability of two different individuals displaying identical genotypes at 1-31 loci in the existing dataset. Thereafter the minimum number of loci displaying identical genotypes between two independent samples to regard them as a true duplicate was computed, in order to have a low probability of accepting a false positive that occurred by chance.

To estimate the probability of getting a false positive match in the current dataset, the 31 loci were ranked from having the most frequent to the least frequent main allele, i.e., from the least informative to the most informative locus for this dataset. The cumulative probability and expected number of individuals sharing N identical loci was estimated by assuming that individuals which are identical at N loci by chance would always share the most common allele of the least informative loci (Suppl. Table 2). This approach was used as it is less computationally intensive than estimating every allelic combination associated with a respective allelic frequency and is also expected to give a more conservative estimation of threshold values that identify true duplicated individuals.

From the procedure above, the minimum number of matching loci between a pair of samples that minimises the risk of false positives was identified as 21 loci (expected number of false positives with 21 matching loci was less than 2 in the current analysis and data set, see Suppl. Table 2). Thus, the number of missing and/or non-matching loci between a pair of individuals that matched at 21 loci could be between zero and ten. Of these individuals with 21 identical genotypes or more, the number of mismatched loci for a pair are shown in Suppl. Figure 2. Most of the pairs of individuals that matched at >21 loci displayed zero or one mismatch, while fifteen displayed five mismatches or more. It is worth noting that no pairs displayed 3 or 4 mismatches, making pairs with five or more mismatches likely candidates for being false positives. Pairs with five or more mismatches were manually checked for biological data and were rejected as true duplicates due to conflicting biological data (e.g., incoherent weight between captures).

Additionally to the procedure above, and to avoid overlooking potential individuals represented twice within the dataset, a second search was done with the threshold set to a minimum of 16 matching loci. A maximum of 20 matching loci was also enforced in this specific procedure to avoid detecting pairs that were identified in the first search described above. With these criteria the number of mismatches could be up to 15, and the number of mismatched loci for a pair are shown in Suppl. Figure 3. More than 300 pairs displayed ten mismatches or more, which contrasts with the fifteen reported in the former step and confirms the minimum threshold of 21 matches as an acceptable solution to avoid false positives. We also observed 15 pairs with less than three mismatches, which were considered to be potentially “true” duplicates. The 15 potential duplicates were verified manually by concordant biological data between captures.


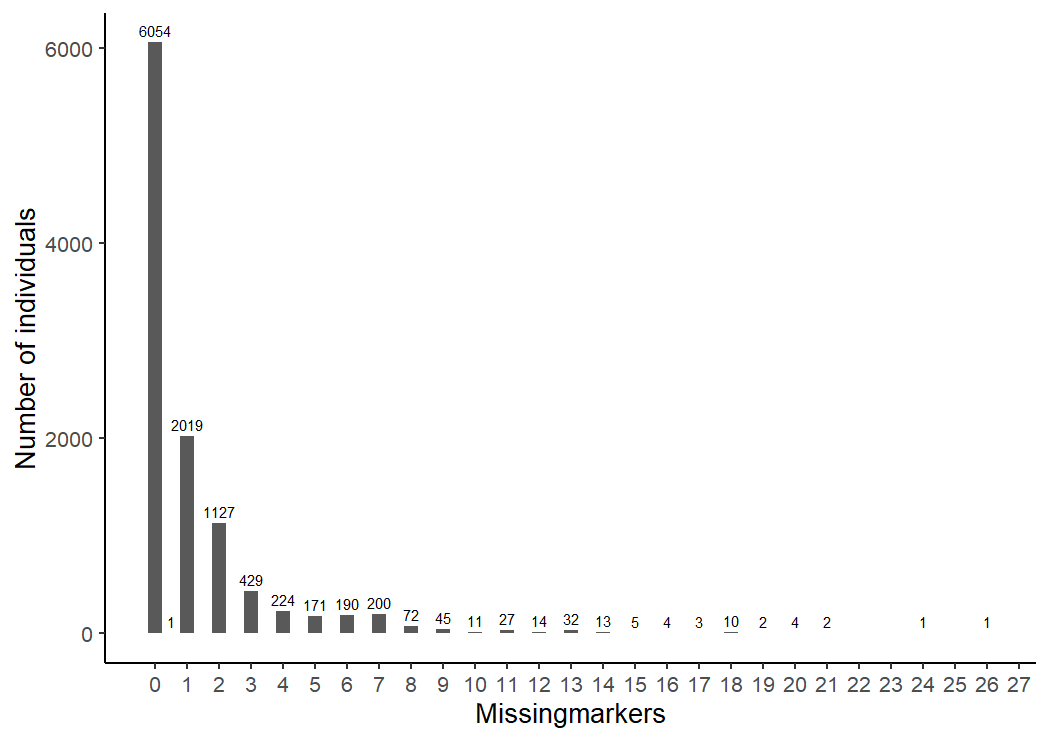


*Figure 1: Number of salmon in the genetic database missing 0 (i.e., data for all 31 markers present) up to 27 markers.*

**Table 1**: *Summary genetic statistics, including number of alleles per locus, observed vs expected heterozygosity, deviations from Hardy-Weinberg equilibrium, and allelic richness, presented by locus.*

| Locus | N alleles | Ho | He | HWE p value | Ar |
| --- | --- | --- | --- | --- | --- |
| SSsp2201 | 32 | 0.94 | 0.94 | 0.000 | 31.7 |
| SSsp2210 | 13 | 0.83 | 0.83 | 0.000 | 12.9 |
| SSspG7 | 24 | 0.88 | 0.89 | 0.000 | 23.9 |
| Ss202 | 18 | 0.8 | 0.81 | 0.000 | 17.9 |
| SsD144 | 34 | 0.94 | 0.94 | 0.000 | 34.0 |
| SsD157 | 37 | 0.93 | 0.94 | 0.000 | 36.9 |
| Sp1605 | 18 | 0.81 | 0.81 | 0.020 | 17.7 |
| Sp2216 | 19 | 0.9 | 0.9 | 0.000 | 19.0 |
| Ss14 | 5 | 0.47 | 0.47 | 0.522 | 4.9 |
| Ss171 | 27 | 0.84 | 0.85 | 0.000 | 27.0 |
| Ss289 | 9 | 0.59 | 0.6 | 0.008 | 8.9 |
| MHC1 | 18 | 0.88 | 0.89 | 0.000 | 17.7 |
| MHC2 | 11 | 0.78 | 0.79 | 0.040 | 10.9 |
| SSsp3016 | 19 | 0.73 | 0.73 | 0.000 | 19.0 |
| SsOsl85 | 21 | 0.86 | 0.87 | 0.011 | 20.9 |
| Ss197 | 28 | 0.91 | 0.92 | 0.009 | 27.9 |
| SsD486 | 5 | 0.04 | 0.04 | 0.127 | 5.0 |
| SsF43 | 11 | 0.71 | 0.7 | 0.001 | 10.7 |
| EST107 | 10 | 0.68 | 0.72 | 0.000 | 9.9 |
| EST19 | 39 | 0.93 | 0.93 | 0.000 | 38.6 |
| EST28 | 11 | 0.44 | 0.44 | 0.931 | 10.7 |
| EST68 | 8 | 0.72 | 0.72 | 0.000 | 8.0 |
| Sleel53 | 7 | 0.28 | 0.28 | 0.939 | 6.9 |
| Sleen82 | 12 | 0.65 | 0.65 | 0.685 | 11.7 |
| SsOSL25 | 15 | 0.73 | 0.73 | 0.000 | 15.0 |
| SsSP2215 | 25 | 0.88 | 0.89 | 0.000 | 24.6 |
| Ss405 | 31 | 0.93 | 0.93 | 0.001 | 30.8 |
| Ss407 | 37 | 0.88 | 0.9 | 0.000 | 37.0 |
| Ss412 | 8 | 0.48 | 0.49 | 0.757 | 7.8 |
| Ss98 | 16 | 0.34 | 0.34 | 0.000 | 16.0 |
| Ssleer15.1 | 7 | 0.56 | 0.57 | 0.015 | 6.8 |

**Table 2**: *Probabilities and expected numbers of individuals displaying identical genotypes by chance at a variable number of markers. Nominal probability: frequency of the most frequent alleles at marker n in all the 10 661 individuals. Cumulative probability: probability that two individuals matches at “n” number of markers. Nr of false positive expected: cumulative number*the number of comparisons (* $\frac{10661*10660}{2}$*).*

| *Number of identical markers* | *Nominal probability* | *Cumulative probability* | *Expected Nr of false positives* |
| --- | --- | --- | --- |
| *1* | *0.9398274* | *0.9398274* | *53403935* |
| *2* | *0.8262827* | *0.7765631* | *44126748* |
| *3* | *0.706641* | *0.5487514* | *31181771* |
| *4* | *0.7021855* | *0.3853253* | *21895388* |
| *5* | *0.6440765* | *0.248179* | *14102306* |
| *6* | *0.632633* | *0.1570062* | *8921584* |
| *7* | *0.5485414* | *0.0861244* | *4893858* |
| *8* | *0.5052059* | *0.04351056* | *2472406* |
| *9* | *0.4759403* | *0.02070843* | *1176718* |
| *10* | *0.4533346* | *0.009387847* | *533446.8* |
| *11* | *0.4360754* | *0.004093809* | *232623.1* |
| *12* | *0.4247256* | *0.001738746* | *98800.97* |
| *13* | *0.3895507* | *0.00067733* | *38487.99* |
| *14* | *0.383313* | *0.000259629* | *14752.95* |
| *15* | *0.3799831* | *9.87E-05* | *5605.871* |
| *16* | *0.3413845* | *3.37E-05* | *1913.757* |
| *17* | *0.273661* | *9.22E-06* | *523.7207* |
| *18* | *0.2611856* | *2.41E-06* | *136.7883* |
| *19* | *0.2358597* | *5.68E-07* | *32.26285* |
| *20* | *0.1863803* | *1.06E-07* | *6.013159* |
| *21* | *0.1850671* | *1.96E-08* | *1.112838* |
| *22* | *0.1840822* | *3.61E-09* | *0.2048536* |
| *23* | *0.1797674* | *6.48E-10* | *0.03682599* |
| *24* | *0.1768127* | *1.15E-10* | *0.006511302* |
| *25* | *0.1663071* | *1.91E-11* | *0.001082876* |
| *26* | *0.1353063* | *2.58E-12* | *0.00014652* |
| *27* | *0.1313198* | *3.39E-13* | *1.92E-05* |
| *28* | *0.1219867* | *4.13E-14* | *2.35E-06* |
| *29* | *0.1002251* | *4.14E-15* | *2.35E-07* |
| *30* | *0.08906294* | *3.69E-16* | *2.10E-08* |
| *31* | *0.07944846* | *2.93E-17* | *1.66E-09* |


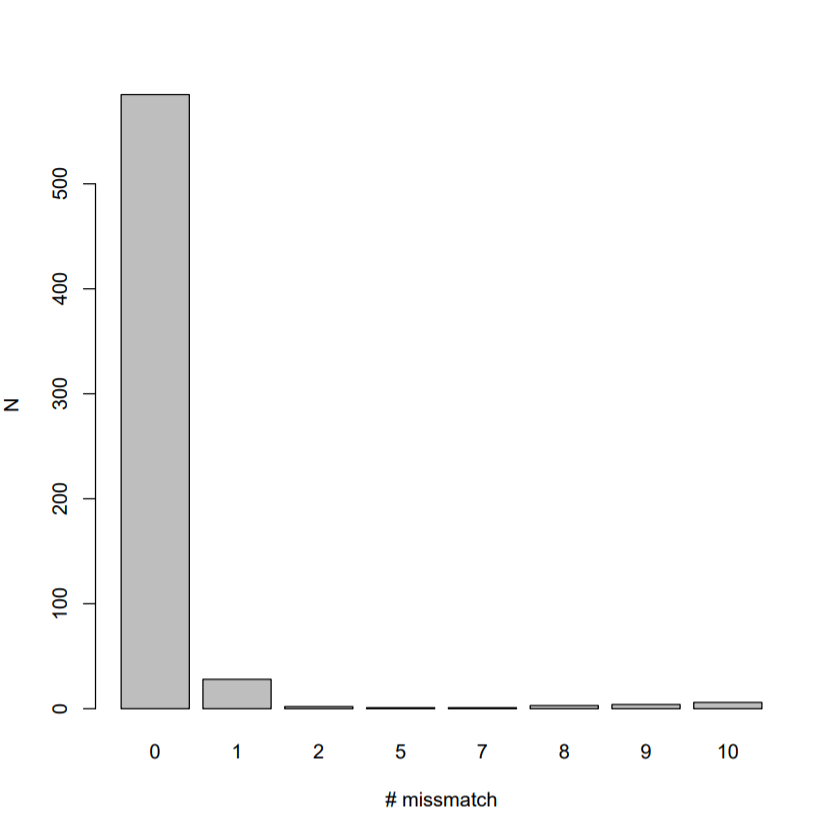


*Figure 2: Distribution of mismatches (i.e., different allele score at the same marker) between pairs of biological samples matching at 21 or more markers.*


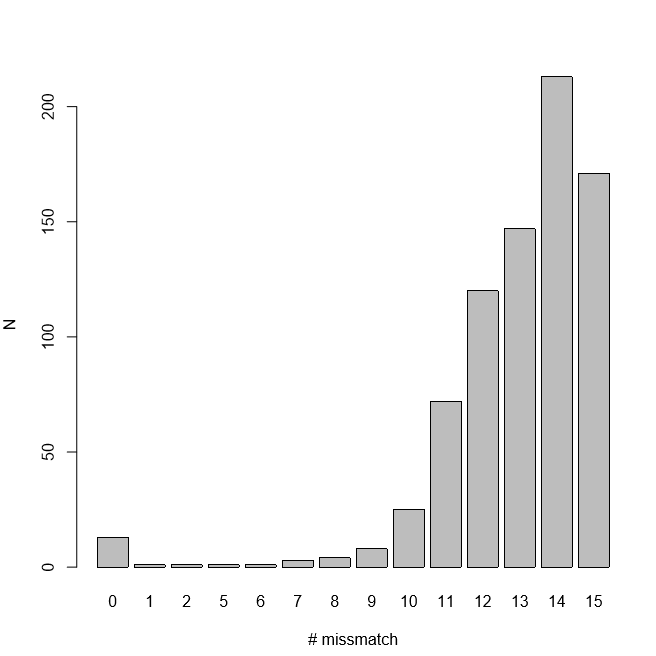


*Figure 3: Distribution of mismatches (i.e., different allele score at the same marker) between pairs of biological samples matching at 16-20 markers.*

Table S1 Two sample chi-square tests comparing the frequency of iteroparity observed (A) using the molecular method versus the scale reading method and (B) comparing the age at first maturity between maiden spawners and future repeat spawners for 2015-2018.

| A | Molecular method | Scale method | Total N | Chi square | Df | P value |
| --- | --- | --- | --- | --- | --- | --- |
| **Total repeat spawners** | **403** | **617** | **8895** | **47.19** | **1** | **0.000** |
| **Total females** | **280** | **381** | **3974** | **16.50** | **1** | **0.000** |
| **Total males** | **117** | **215** | **4609** | **29.40** | **1** | **0.000** |
| Total 1SW | 79 | 76 | 2962 | 0.03 | 1 | 0.871 |
| Total 2SW | 268 | 314 | 4765 | 3.71 | 1 | 0.054 |
| **Total MSW** | **56** | **227** | **1031** | **118.37** | **1** | **0.000** |
| **Total alternative strategy** | **303** | **267** | **362** | **9.30** | **1** | **0.002** |
| **Total consecutive strategy** | **59** | **93** | **362** | **9.30** | **1** | **0.002** |
| **B** |  |  |  |  |  |  |
| 2015 | Future repeat spawner | Maiden spawner | Total N | Chi square | Df | P value |
| **1SW** | **49** | **1072** | **147, 1941** | **25.48** | **1** | **0.000** |
| **2SW** | **80** | **706** | **147, 1941** | **18.20** | **1** | **0.000** |
| MSW | 18 | 163 | 147, 1941 | 2.10 | 1 | 0.148 |
| 2016 |  |  |  |  |  |  |
| **1SW** | **8** | **312** | **119, 2000** | **6.23** | **1** | **0.013** |
| **2SW** | **101** | **1505** | **119, 2000** | **5.16** | **1** | **0.023** |
| MSW | 10 | 183 | 119, 2000 | 0.01 | 1 | 0.912 |
| 2017 |  |  |  |  |  |  |
| **1SW** | **8** | **526** | **58, 1795** | **5.86** | **1** | **0.016** |
| **2SW** | **41** | **916** | **58, 1795** | **7.93** | **1** | **0.005** |
| MSW | 9 | 353 | 58, 1795 | 0.38 | 1 | 0.538 |
| 2018 |  |  |  |  |  |  |
| 1SW | 3 | 506 | 6, 1492 | 0.16 | 1 | 0.690 |
| 2SW | 2 | 861 |  | 0.63 | 1 | 0.428 |
| MSW | 1 | 125 |  | 0.00 | 1 | 1.000 |

Table S2: Bonferroni-adjust post-hoc tests of the significant terms with more than 2 levels in model 1.1. comparing the frequency of iteroparity within (A) years, (B) sea age at first maturity, (C) sex within sea age at first maturity, (D) spawning strategy within sea age at first maturity, (E) sea age at first maturity within years.

| A Contrast |  |  | Ratio | Standard error | Df | T ratio | P value |
| --- | --- | --- | --- | --- | --- | --- | --- |
| 2015 | - | 2016 | 0.77 | 0.28 | 33 | -0.72 | 0.951 |
| **2015** | **-** | **2017** | **0.27** | **0.09** | **33** | **-3.92** | **0.004** |
| 2015 | - | 2018 | 0.52 | 0.18 | 33 | -1.89 | 0.341 |
| 2015 | - | 2019 | 0.77 | 0.28 | 33 | -0.72 | 0.952 |
| **2016** | **-** | **2017** | **0.35** | **0.11** | **33** | **-3.36** | **0.016** |
| 2016 | - | 2018 | 0.67 | 0.23 | 33 | -1.17 | 0.767 |
| 2016 | - | 2019 | 1.01 | 0.35 | 33 | 0.02 | 1.000 |
| 2017 | - | 2018 | 1.94 | 0.58 | 33 | 2.19 | 0.210 |
| **2017** | **-** | **2019** | **2.89** | **0.91** | **33** | **3.37** | **0.015** |
| 2018 | - | 2019 | 1.50 | 0.48 | 33 | 1.25 | 0.723 |
| B Contrast |  |  | Ratio | Standard error | Df | T ratio | P value |
| **1SW** | **-** | **2SW** | **0.29** | **0.09** | **33** | **-3.85** | **0.002** |
| 1SW | - | MSW | 1.69 | 0.76 | **33** | 1.16 | 0.488 |
| **2SW** | **-** | **MSW** | **5.87** | **2.27** | **33** | **4.58** | **0.000** |
| C Contrast |  |  | Ratio | Standard error | Df | T ratio | P value |
| **1 SW females** | **-** | **1SW males** | **0.09** | **0.04** | **33** | **-4.81** | **0.000** |
| **2SW females** | **-** | **2SW males** | **3.98** | **1.23** | **33** | **4.45** | **0.000** |
| **MSW females** | **-** | **MSW males** | **13.36** | **8.73** | **33** | **3.97** | **0.000** |
| D Contrast |  |  | Ratio | Standard error | Df | T ratio | P value |
| 1SW Alternate | - | 1SW Consecutive | 1.46 | 0.58 | 33 | 0.96 | 0.346 |
| **2SW Alternate** | **-** | **2SW Consecutive** | **7.14** | **2.22** | 33 | **6.32** | **0.000** |
| **MSW Alternate** | **-** | **MSW Consecutive** | **5.33** | **2.72** | 33 | **3.72** | **0.003** |
| E 2015 |  |  | Ratio | Standard error | Df | T ratio | P value |
| **1SW** | **-** | **2SW** | 0.2045 | 0.1711 | 33 | -1.897 | 0.1556 |
| 1SW | - | MSW | 0.3304 | 0.3034 | 33 | -1.206 | 0.458 |
| **2SW** | **-** | **MSW** | 1.6159 | 0.9637 | 33 | 0.805 | 0.7028 |
| 2016 |  |  | Ratio | Standard error | Df | T ratio | P value |
| **1SW** | **-** | **2SW** | 0.5207 | 0.3438 | 33 | -0.988 | 0.5894 |
| 1SW | - | MSW | 2.8639 | 2.4298 | 33 | 1.24 | 0.4386 |
| **2SW** | **-** | **MSW** | 5.4996 | 4.1237 | 33 | 2.273 | 0.0738 |
| 2017 |  |  | Ratio | Standard error | Df | T ratio | P value |
| **1SW** | **-** | **2SW** | 0.8941 | 0.3637 | 33 | -0.275 | 0.9591 |
| 1SW | - | MSW | 7.8559 | 4.8626 | 33 | 3.33 | 0.0059 |
| **2SW** | **-** | **MSW** | 8.7868 | 5.1041 | 33 | 3.741 | 0.002 |
| 2018 |  |  | Ratio | Standard error | Df | T ratio | P value |
| **1SW** | **-** | **2SW** | 0.0933 | 0.0543 | 33 | -4.072 | 0.0008 |
| 1SW | - | MSW | 1.1032 | 0.8678 | 33 | 0.125 | 0.9914 |
| **2SW** | **-** | **MSW** | 11.8246 | 6.9714 | 33 | 4.19 | 0.0006 |
| 2019 |  |  | Ratio | Standard error | Df | T ratio | P value |
| **1SW** | **-** | **2SW** | 0.2199 | 0.1409 | 33 | -2.363 | 0.0609 |
| 1SW | - | MSW | 1.6612 | 1.4205 | 33 | 0.594 | 0.8245 |
| **2SW** | **-** | **MSW** | 7.5531 | 4.9175 | 33 | 3.106 | 0.0106 |

Table S3: Bonferroni-adjust post-hoc tests of the significant terms with more than 2 levels in model 1.2 investigating size differences between maiden and second spawning for (A) years, (B) sea age at first maturity, (C) spawning strategy within sea age at first maturity.

| A Contrast |  |  | Ratio | Standard error | Df | T ratio | P value |
| --- | --- | --- | --- | --- | --- | --- | --- |
| 2013 | - | 2014 | -1.33 | 1.94 | 323 | -0.69 | 0.959 |
| 2013 | - | 2015 | -3.54 | 1.98 | 323 | -1.79 | 0.382 |
| 2013 | - | 2016 | -5.12 | 2.09 | 323 | -2.46 | 0.104 |
| 2013 | - | 2017 | -4.32 | 1.84 | 323 | -2.34 | 0.134 |
| 2014 | - | 2015 | -2.21 | 2.31 | 323 | -0.96 | 0.874 |
| 2014 | - | 2016 | -3.79 | 2.37 | 323 | -1.60 | 0.496 |
| 2014 | - | 2017 | -2.99 | 2.05 | 323 | -1.46 | 0.590 |
| 2015 | - | 2016 | -1.58 | 2.67 | 323 | -0.59 | 0.976 |
| 2015 | - | 2017 | -0.78 | 1.88 | 323 | -0.42 | 0.994 |
| 2016 | - | 2017 | 0.80 | 2.29 | 323 | 0.35 | 0.997 |
| B Contrast |  |  | Ratio | Standard error | Df | T ratio | P value |
| **1SW** | **-** | **2SW** | **5.36** | **1.57** | **323** | **3.42** | **0.002** |
| **1SW** | **-** | **MSW** | **10.71** | **2.29** | **323** | **4.69** | **0.000** |
| 2SW | - | MSW | 5.35 | 2.68 | 323 | 2.00 | 0.114 |
| C Contrast |  |  | Ratio | Standard error | Df | T ratio | P value |
| **1SW Alternate** | **-** | **1SW Consecutive** | **15.14** | **1.22** | **323** | **12.39** | **0.000** |
| **2SW Alternate** | **-** | **2SW Consecutive** | **9.36** | **0.97** | **323** | **9.68** | **0.000** |
| **MSW Alternate** | **-** | **MSW Consecutive** | **7.02** | **1.90** | **323** | **3.70** | **0.000** |

Table S4: Bonferroni-adjust post-hoc tests of the significant terms with more than 2 levels in model 1.3a investigating difference in the day of river entry between maiden and second spawning within maiden year of entry for alternate repeat spawners.

| Contrast |  |  | Ratio | Standard error | Df | T ratio | P value |
| --- | --- | --- | --- | --- | --- | --- | --- |
| 2013 | - | 2014 | 1.1 | 0.0842 | 269 | 1.241 | 0.7271 |
| 2013 | **-** | 2015 | 1.159 | 0.0734 | 269 | 2.33 | 0.1385 |
| 2013 | - | 2016 | 0.996 | 0.0741 | 269 | -0.058 | 1 |
| 2013 | - | 2017 | 1.077 | 0.0711 | 269 | 1.128 | 0.7916 |
| 2014 | **-** | 2015 | 1.054 | 0.055 | 269 | 1.01 | 0.8509 |
| 2014 | - | 2016 | 0.905 | 0.0587 | 269 | -1.532 | 0.5423 |
| 2014 | - | 2017 | 0.98 | 0.0539 | 269 | -0.373 | 0.9959 |
| **2015** | **-** | **2016** | **0.859** | **0.0425** | **269** | **-3.071** | **0.0198** |
| 2015 | **-** | 2017 | 0.929 | 0.0333 | 269 | -2.045 | 0.2476 |
| 2016 | - | 2017 | 1.082 | 0.0561 | 269 | 1.519 | 0.5509 |

Table S5: S1 Two sample chi-square tests comparing the frequency of female and male kelts observed within each year of capture.

|  | Female | Male | Total N | Chi square | Df | P value |
| --- | --- | --- | --- | --- | --- | --- |
| **2018 kelts** | **37** | **10** | **47** | **28.77** | **1** | **0.000** |
| **2019 kelts** | **45** | **12** | **57** | **35.93** | **1** | **0.000** |
| **2020 kelts** | **38** | **3** | **41** | **56.39** | **1** | **0.000** |
